# Construction and verification of an endoplasmic reticulum stress-related prognostic model for endometrial cancer based on WGCNA and machine learning algorithms

**DOI:** 10.3389/fonc.2024.1362891

**Published:** 2024-04-25

**Authors:** Shanshan Lin, Changqiang Wei, Yiyun Wei, Jiangtao Fan

**Affiliations:** ^1^ Department of Obstetrics and Gynecology, The First Affiliated Hospital of Guangxi Medical University, Nanning, Guangxi, China; ^2^ Department of Prenatal Diagnosis, The First Affiliated Hospital of Guangxi Medical University, Nanning, Guangxi, China

**Keywords:** endometrial cancer, endoplasmic reticulum stress, machine learning, WGCNA, prognostic model

## Abstract

**Background:**

Endoplasmic reticulum (ER) stress arises from the accumulation of misfolded or unfolded proteins within the cell and is intricately linked to the initiation and progression of various tumors and their therapeutic strategies. However, the precise role of ER stress in uterine corpus endometrial cancer (UCEC) remains unclear.

**Methods:**

Data on patients with UCEC and control subjects were obtained from The Cancer Genome Atlas (TCGA) and Gene Expression Omnibus (GEO) databases. Using differential expression analysis and Weighted Gene Co-expression Network Analysis (WGCNA), we identified pivotal differentially expressed ER stress-related genes (DEERGs). Further validation of the significance of these genes in UCEC was achieved through consensus clustering and bioinformatic analyses. Using Cox regression analysis and several machine learning algorithms (least absolute shrinkage and selection operator [LASSO], eXtreme Gradient Boosting [XGBoost], support vector machine recursive feature elimination [SVM-RFE], and Random Forest), hub DEERGs associated with patient prognosis were effectively identified. Based on the four identified hub genes, a prognostic model and nomogram were constructed. Additionally, a drug sensitivity analysis and *in vitro* validation experiments were performed.

**Results:**

A total of 94 DEERGs were identified in patients with UCEC and healthy controls. Consensus clustering analysis revealed significant differences in prognosis, typical immune checkpoints, and tumor microenvironments between the subtypes. Using Cox regression analysis and machine learning, four hub DEERGs, MYBL2, RADX, RUSC2, and CYP46A1, were identified to construct a prognostic model. The reliability of the model was validated using receiver operating characteristic (ROC) curves. Decision curve analysis (DCA) demonstrated the superior predictive ability of the nomogram in terms of 3- and 5-year survival, compared with that of other clinical indicators. Drug sensitivity analysis revealed increased sensitivity to dactinomycin, docetaxel, selumetinib, and trametinib in the low-risk group. The expressions of RADX, RUSC2, and CYP46A1 were downregulated, whereas that of MYBL2 was upregulated in UCEC tissues, as demonstrated by reverse transcription-quantitative polymerase chain reaction (RT-qPCR) and immunofluorescence assays.

**Conclusion:**

This study developed a stable and accurate prognostic model based on multiple bioinformatics analyses, which can be used to assess the prognosis of UCEC. This model may contribute to future research on the risk stratification of patients with UCEC and the formulation of novel treatment strategies.

## 1 Introduction

Uterine corpus endometrial cancer (UCEC) is one of the most prevalent and lethal gynecological malignancies worldwide. The global incidence of UCEC in 2020 was approximately 417,000, with a mortality rate of approximately 97,000. Furthermore, UCEC is the sixth most common cancer in women (1). In the United States alone, 66,200 new UCEC cases and 13,030 deaths are projected to be reported for 2023 (2). Chinese cancer statistics indicate that UCEC is the second most prevalent gynecological malignant tumor (3). The primary treatment modality for UCEC is comprehensive surgery including total hysterectomy and bilateral salpingo-oophorectomy (4). The latest National Comprehensive Cancer Network® (NCCN) Clinical Practice Guidelines for Uterine Oncology (2023) suggest that the preferred treatment regimen for endometrial cancer is carboplatin combined with paclitaxel. Trastuzumab is also required in patients with advanced or recurrent HER2-positive disease. Other recommended regimens include carboplatin/docetaxel and carboplatin/paclitaxel/bevacizumab (5). The 5-year relative survival rate of patients with UCEC across all stages is approximately 81%. Nevertheless, despite advancements in UCEC treatment, the prognosis of patients with distant metastasis remains poor, with a 5-year relative survival rate as low as 17% (6). The low survival rates reflect the limited availability of effective treatment options for patients with recurrent and metastatic UCEC. Hence, the identification of novel and effective biomarkers is of paramount importance for targeted therapy and prognostic assessment of UCEC.

The endoplasmic reticulum (ER) is a multifaceted organelle responsible for the synthesis and modification of proteins, anabolic steroids, and lipids. Under stress conditions, such as hypoxia, nutrient deprivation, oxidative stress, lactic acidosis, and Ca^2+^ depletion, substantial accumulation of unfolded and misfolded proteins occurs in the ER lumen, intensifying the load on the ER and inducing stress (7). Mild ER stress can reinstate homeostasis via signal sensors, facilitating cellular adaptation, while severe ER stress can activate inflammasomes and apoptosis-associated genes, resulting in cell death (8). ER stress has been intricately linked to the onset and progression of malignant tumors including glioblastoma, multiple myeloma, breast cancer, gastric cancer, esophageal cancer, and liver cancer (9, 10). Sustained, high-level activation of ER signaling sensors (PERK, ATF6, and IRE1α) was detectable in these cancer tissues, which may promote tumor cell proliferation, migration, invasion, vascularization, drug resistance, and immunosuppression (11).

Abnormal activation of ER stress sensors and their downstream signaling pathways is a crucial regulator of cancer development, metastasis, and response to various treatments (8). Ongoing research is investigating the relationship between ER stress and UCEC. ER stress-related proteins are reported to be closely associated with apoptosis in UCEC (12). Another study suggests that transcription factors induced by ER stress can promote the growth of UCEC through macrophage recruitment (13). Furthermore, ER stress is closely associated with the promotion of UCEC proliferation, invasion, and chemical resistance (14).

In summary, these findings indicate that ER stress plays a crucial role in UCEC, and ER stress intervention is expected to be a novel clinical treatment direction in the future. Consequently, comprehensive exploration of the relationship between ER stress and UCEC is imperative.

## 2 Materials and methods

### 2.1 Datasets and patient selection

**Figure 1:**
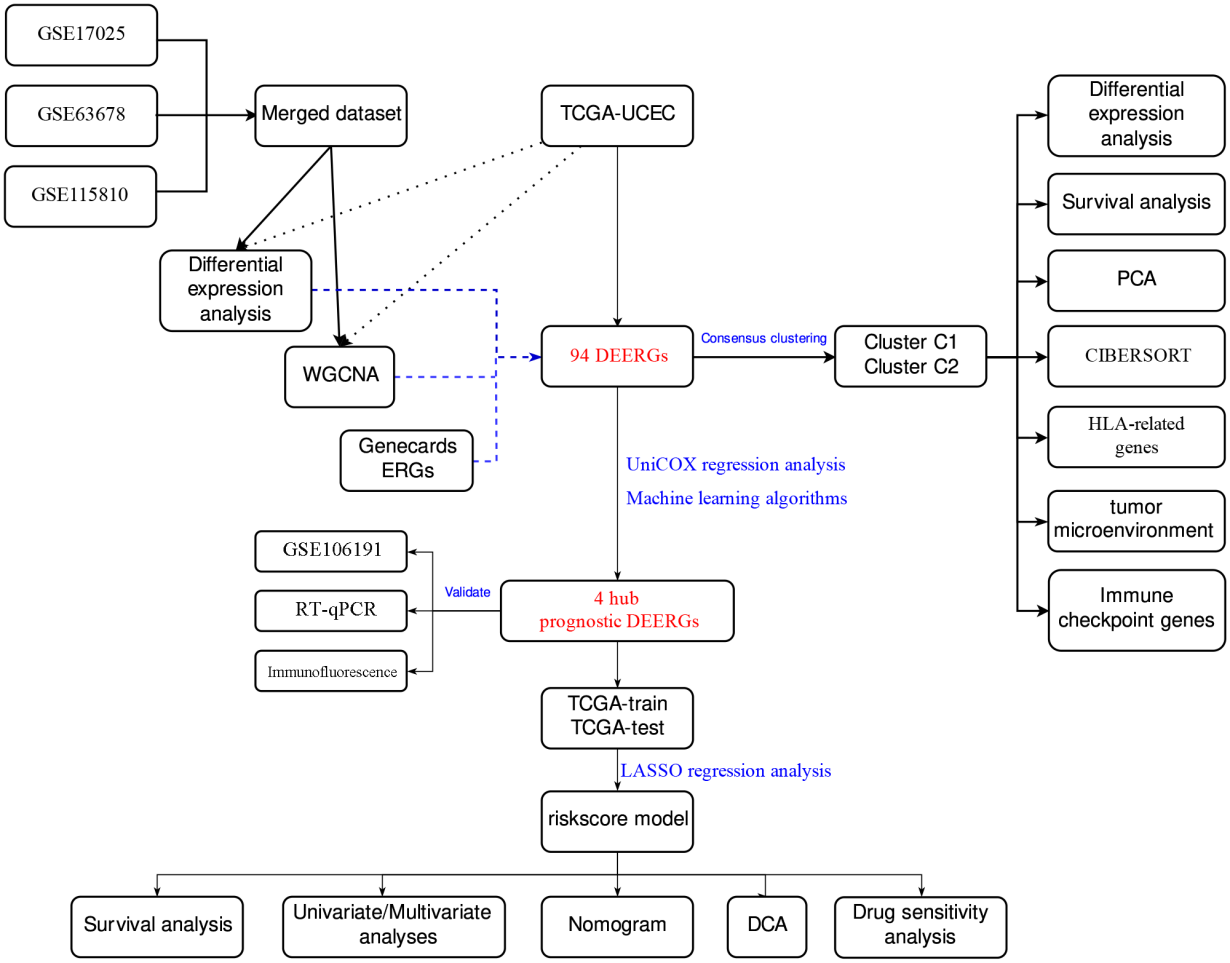
Main flowchart of study design.

A flowchart of this study is shown in **Figure 1**. We acquired TCGA-UCEC from The Cancer Genome Atlas (TCGA) database and GSE17025, GSE63678, GSE115810, and GSE106191 from the Gene Expression Omnibus (GEO) database. GSE17025, GSE63678, and GSE115810 were merged into a new dataset for further analysis, and GSE106191 served as an external validation set (**Table 1**). ER stress-related genes (ERGs) were obtained from the GeneCards database (https://www.genecards.org/). ERGs with relevance scores of >5 were chosen for this study, and comprehensive information on these genes is provided in **Supplementary Table 1**.

**Table 1:** Baseline characteristics of GEO datasets used in this study.

Dataset	Platforms	Sample size
GSE17025	GPL570	103 (91 UCEC samples and 12 control samples)
GSE63678	GPL571	12 (7 UCEC samples and 5 control samples)
GSE115810	GPL96	27 (24 UCEC samples and 3 control samples)
GSE106191	GPL570	97 (64 UCEC samples and 33 control samples)
TCGA-UCEC	TCGA	579 (544 UCEC samples and 35 control samples)

Next, 20 UCEC and 20 normal endometrial tissue samples were obtained from the Department of Obstetrics and Gynecology of the First Affiliated Hospital of Guangxi Medical University. The UCEC stage was assessed according to the Federation International of Gynecology and Obstetrics (FIGO) 2009 guidelines, and the diagnosis was made by experienced pathologists. Normal endometrial tissues obtained from patients who underwent hysterectomies for unrelated endometrial diseases were used as controls. None of the patients received systemic therapy before surgery. The clinical data of patients diagnosed with UCEC are presented in **Table 2**. The study adhered to the principles of the Declaration of Helsinki, and ethical approval was obtained from the Research Ethics Committee of the First Affiliated Hospital of Guangxi Medical University (No. 2023-S033-01). All the patients included in the study provided written informed consent before surgery.

**Table 2:** The clinical data of patients diagnosed with UCEC.

Clinical parameters		N
Age	<60	13
	≥60	7
Grade	G1-G2	14
	G3-G4	6
Differentiation	Low and Middle	12
	High	8
Invasion depth	Superficial	6
	Deep	14
Lymphatic metastasis	No	15
	Yes	5
Vascular invasion	No	19
	Yes	1
Distal metastasis	No	20
	Yes	0

### 2.2 Differential expression analysis and co-expression network analysis

Differential expression analysis was performed using the “limma” R package (15). For the TCGA-UCEC dataset, the screening criterion was set to |log2 FC| > 2, and the adjusted p-value was <0.05. For the GEO dataset, the “sva” and “limma” R packages were used to perform data merging and batch correction for GSE17025, GSE63678, and GSE115810. The screening criterion for differential expression analysis was |log2 FC| > 1, and the adjusted p-value was <0.05.

The “WGCNA” R software package was used to construct the co-expression network for the TCGA-UCEC and merged GEO data sets (16). This approach demonstrated the correlation of gene networks with clinical features. The minimum number of modular genes was set to 90, and the genes were clustered to categorize them into distinct modules. Finally, the clinical information was integrated with the modules, and genes in the top three modules that were most relevant to UCEC were selected for subsequent analysis. The results of the gene differential expression analysis and Weighted Gene Co-expression Network Analysis (WGCNA) intersected with the ERGs, and common genes were identified as differentially expressed ER stress-related genes (DEERGs).

### 2.3 Consensus clustering

To identify UCEC subtypes related to DEERGs, consensus clustering was performed through the “ConsensusClusterPlus” R package (17). To ensure the robustness and reproducibility of the results, 1000 repetitions were executed, and the PACmethod was used to determine the optimal number of clusters within the range of k = 2–10. The outcomes of this process are represented through clear cluster plots using the pheatmap function in the R software.

Subsequently, the “limma” package was employed to conduct principal component analysis (PCA) and differential expression analysis between clusters. This method facilitated clear visualization of the sample distribution. The outcomes of the inter-cluster analysis were then visually presented using the “scatterplot3d,” “reshape2,” and “ggpubr” packages.

Immune cell infiltration analysis was performed using the CIBERSORT algorithm to assess differences in the infiltration levels of 22 immune cells between clusters (18). The conditions were set to perm = 1000 and p < 0.05, and the results were visualized via the “vioplot” package.

Differential expression analysis of several typical immune checkpoint genes and human leukocyte antigen (HLA)-related genes was performed using the “limma” package. This process revealed the distinct expression patterns of immune checkpoints and HLA-related genes among the different clusters. Lastly, we evaluated the tumor microenvironment (TME) with the “estimate” package, offering robust support for a comprehensive understanding of UCEC characteristics.

### 2.4 Cox regression model and machine learning

To identify DEERGs with prognostic value, the correlation between each gene and survival status in the TCGA-UCEC dataset was initially evaluated using Cox regression modeling. Patients with survival times of <30 days were excluded to enhance reliability and robustness. For prognosis-related DEERGs, four machine learning algorithms were used to screen for genes that were important to UCEC. Least absolute shrinkage and selection operator (LASSO) regression analysis employed the “glmnet” package (19), while support vector machine recursive feature elimination (SVM-RFE) analysis utilized the “e1071,” “kernlab,” and “caret” packages (20). Random Forest (21) and eXtreme Gradient Boosting (XGBoost) (22) algorithms were executed via the “randomForest” and “xgboost” packages, respectively. The results of the four algorithms were intersected, and common genes were identified as hub DEERGs. Prognostic impact was analyzed using K-Mplotter curves, and hub gene expression was validated using the external validation set GSE106191.

### 2.5 Construction and validation of the risk score model

Utilizing the “caret” package, the TCGA-UCEC dataset was randomly partitioned into a training set and a test set at a 7:3 ratio. Subsequently, a prognostic model was constructed in the TCGA-train set based on the expression of hub DEERGs using LASSO Cox regression, and the risk score was calculated as follows: risk score = ∑ (Xi * Yi) (X: coefficient, Y: gene expression level). TCGA-train patients were stratified into low- and high-risk groups according to the median risk score, and Kaplan-Meier analysis was used to compare overall survival (OS) between the two groups.

To further validate the performance of the model, receiver operating characteristic (ROC) curve analyses were performed at 1, 3, and 5 years using the “survival,” “survminer,” and “timeROC” R packages (23). The TCGA test and data were used for model validation. In this validation process, gene expression normalization was achieved through the “scale” function, and risk scores were computed using the same formula as that in the TCGA-train cohort. The patients in the validation set were similarly categorized into low- or high-risk groups to confirm the accuracy of the model.

### 2.6 Independent prognostic analysis

We acquired the clinical information (age, tumor stage, grade, and BMI) of patients in the TCGA-UCEC cohort, which was considered potentially relevant to patient survival status. Subsequently, this information was integrated with the risk score in our regression model for a comprehensive analysis. Using the Kaplan-Meier “survival” package, a univariate Cox regression model analysis was initially performed. The association of each clinical variable with OS was individually assessed to evaluate their independent roles in survival prognosis. Next, a multivariate Cox regression model analysis that considered all relevant clinical variables was performed. This helped determine the independent effect of each variable on survival prognosis while considering other factors. Thus, we gained a more comprehensive understanding of the importance of each clinical variable, which contributes to more precise prognostic information.

### 2.7 Construction of the nomogram and decision curve analysis

Based on the “rms” package, a nomogram was generated to visualize the results of the Cox regression model. The nomogram demonstrated the prognostic value of various clinical features and risk scores. Additionally, utilizing the “ggDCA” package (24), a decision curve analysis (DCA) was constructed to evaluate optimal decision strategies. As a straightforward method for evaluating the effectiveness of clinical prediction models, this may help determine the practical clinical utility of the models across various thresholds and patient populations.

### 2.8 Drug sensitivity analyses

The “oncoPredict” package (25) was used to perform drug sensitivity analysis to identify potential valuable drugs for treating distinct risk groups of UCEC, thereby advancing the development of individualized treatment approaches. This analysis was based on the GDSC database and obtained half-maximal inhibitory concentration (IC50) values for each drug based on gene expression data from the UCEC. To improve the accuracy of the analysis, a threshold of 10 was set for the minimum number of samples, and genes with small fluctuations in gene expression were excluded.

### 2.9 Reverse transcription-quantitative polymerase chain reaction

Total RNA was extracted from the tissues using TRIzol reagent (Takara, Japan). The RNA was reverse-transcribed into cDNA. PCR was performed using the SYBR Green Master Mix kit (Qiagen, Germany) with cDNA as the template and glyceraldehyde 3-phosphate dehydrogenase (GAPDH) as the internal reference. Primers were designed and synthesized by Sangon Biotech Co., Ltd. (China), and the primer sequences are listed in **Table 3**. The relative expression of hub gene mRNA was calculated by the 2-^ΔΔ^CT method, and during reverse transcription-quantitative polymerase chain reaction (RT-qPCR), primer specificity was ensured by observing the melting curves of the reactions. The experiment was conducted with at least three technical replicates. In each replicate well of the sample, a difference in computed tomography (CT) values of <0.5 was considered eligible for analysis. Finally, an unpaired t-test was used to assess the differences in the relative mRNA expression levels between the two groups.

**Table 3:** The primers of hub DEERGs and GAPDH.

Gene name	Primer orientation	Sequences
MYBL2	Forward	CTTGAGCGAGTCCAAAGACTG
	Reverse	AGTTGGTCAGAAGACTTCCCT
CYP46A1	Forward	GCCGTGTGCTCCAAGATGTG
	Reverse	GAACTTCTTAACCGACTCAGGACTC
RADX	Forward	TTCAGGCACAGTGTCAGTGATTATG
	Reverse	GAAGAACTAAACCAACCCGCAAAC
RUSC2	Forward	GAGTGTGGTTGAGGCTTCC
	Reverse	GGGTCTGGATGATGTCTTCG
GAPDH	Forward	CAGGAGGCATTGCTGATGAT
	Reverse	GAAGGCTGGGGCTCATTT

### 2.10 Immunofluorescence assay

First, the samples were prepared into 3-μm slides and deparaffinized three times in xylene for 10 min each time. They were then rehydrated three times in 100% ethanol for 10 min and 5 min each time. Subsequent dehydration was sequentially performed in 95%, 85%, and 75% ethanol for 5 min each. Antigen retrieval sodium citrate (pH 6.0) was then added to the microwaveable vessel. The contents were boiled for 15 min in a microwave oven (1200 W) at moderate heat. Next, 3% hydrogen peroxide (H_2_O_2_) was added, and the cells were incubated for 15 min in the dark at room temperature to block endogenous peroxidases. For blocking, a 3% Bovine Serum Albumin (BSA) solution (Solarbio, China) was added to cover the tissues, which were then incubated for 30 min at room temperature. Tissues with the primary antibody (MYBL2, Abcam, UK; RADX, abmart, China; CYP46A1, Abcam, UK; RUSC2, Thermo Fisher, USA) were incubated in a humidified chamber overnight at 4 °C. The tissues were then incubated with a secondary antibody (labeled with fluorochrome) for 1 h at room temperature in the dark. DAPI (4’,6-diamidino-2-phenylindole) solution (Solarbio) was added to the samples, which were incubated in the dark at room temperature for 10 min. Finally, the slides were washed three times with phosphate-buffered saline (PBS) for 5 min each.

### 2.11 Statistical analysis

Statistical analysis was performed using R software (version 4.3.0). The “ggplot2” and “ggpubr” packages were utilized for data visualization. For normally distributed continuous variables, analysis was performed using Student’s t-test, while the Wilcoxon test was employed to compare the mRNA expression levels (non-normally distributed variables) of individual genes between the UCEC and control groups. Unless otherwise stated, a p-value of <0.05 was considered indicative of statistical significance in all analyses.

## 3 Results

### 3.1 Differential expression of ERGs between tumor and normal tissues

**Figure 2:**
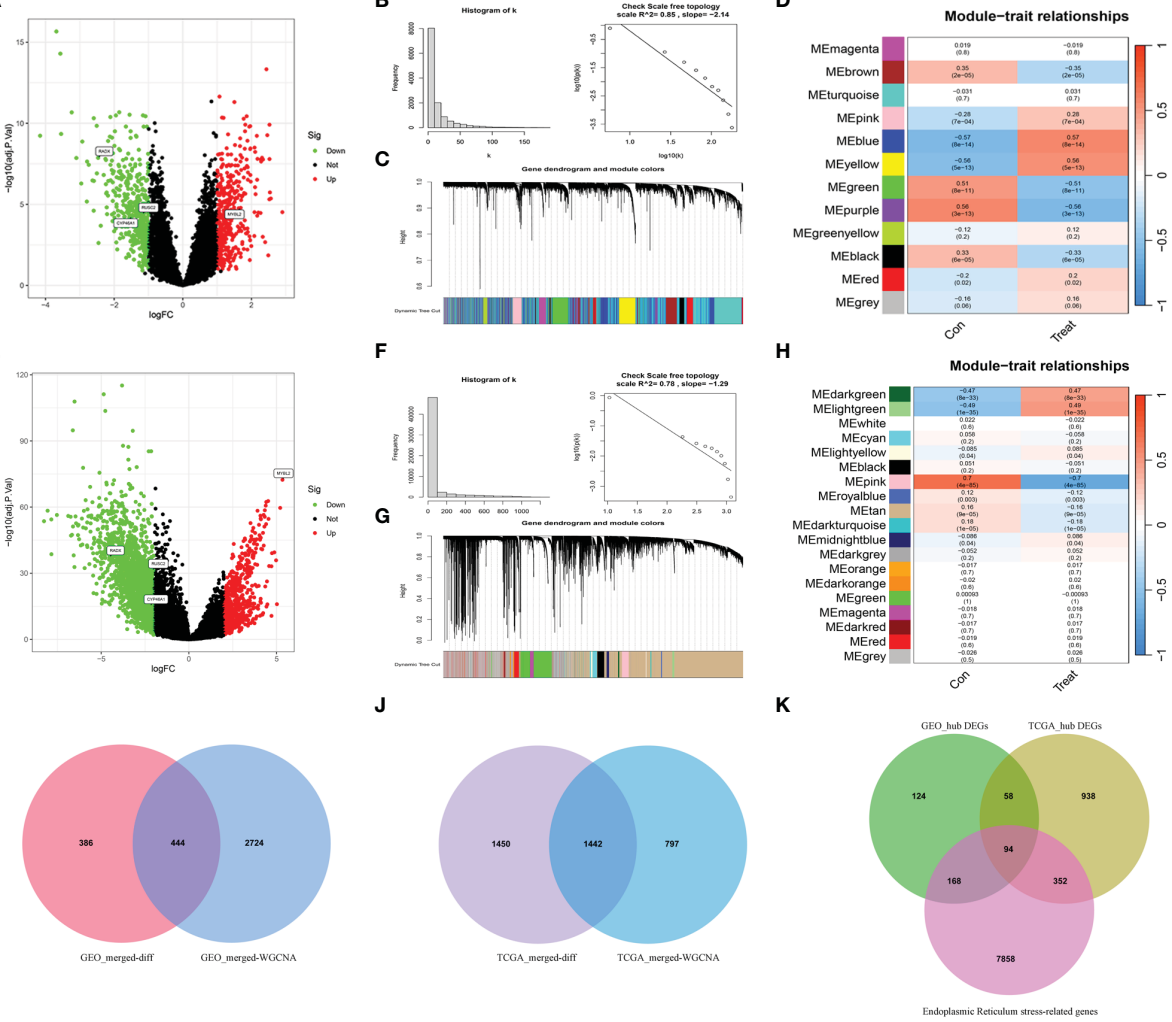
The results of differential expression analysis and WGCNA. **(A, E)** The volcano plots of DEGs in GEO and TCGA datasets, respectively. The red dots represent the up-regulated genes and the green dots represent the down-regulated genes, while gray dots represent nonsignificant genes. **(B, F)** The histograms of connectivity distribution and the scale-free topology in GEO and TCGA datasets, respectively. **(C, G)** The dendrograms of genes clustered via the dissimilarity measure in GEO and TCGA datasets, respectively. **(D, H)** The heatmaps of the correlation between genes and clinical traits in GEO and TCGA datasets, respectively. **(I, J)** The Venn diagrams of overlapping genes between DEGs and the genes of the hub module in GEO and TCGA datasets, respectively. **(K)** Venn diagram showed the overlapping genes between hub DEGs in the GEO dataset, ERGs, and hub DEGs in the TCGA dataset.

Differential expression analysis was conducted on the merged Gene Expression Omnibus (GEO) dataset to identify 830 differentially expressed genes (DEGs) (**Figure 2A**). Subsequently, WGCNA was conducted on the dataset, with β=5 (R^2 ^= 0.85) selected as the soft threshold to construct a scale-free network (**Figures 2B, C**), and 12 modules were successfully screened (**Figure 2D**). The intersection of DEGs with genes in the selected modules resulted in the identification of 444 hub DEGs (**Figure 2I**). A similar analysis was carried out on TCGA-UCEC dataset, revealing 2892 DEGs, compared with normal tissues (**Figure 2E**). In WGCNA, β=7 (R^2^ = 0.78) was selected to construct a scale-free network (**Figures 2F, G**), and 19 modules were identified (**Figure 2H**). Finally, 1442 hub DEGs were identified (**Figure 2J**). By overlapping the hub DEGs from both TCGA and GEO analyses, along with the ER stress-related genes (ERGs), 94 DEERGs were obtained (**Figure 2K**).

### 3.2 Identification of ERG-related subtypes in UCEC

**Figure 3:**
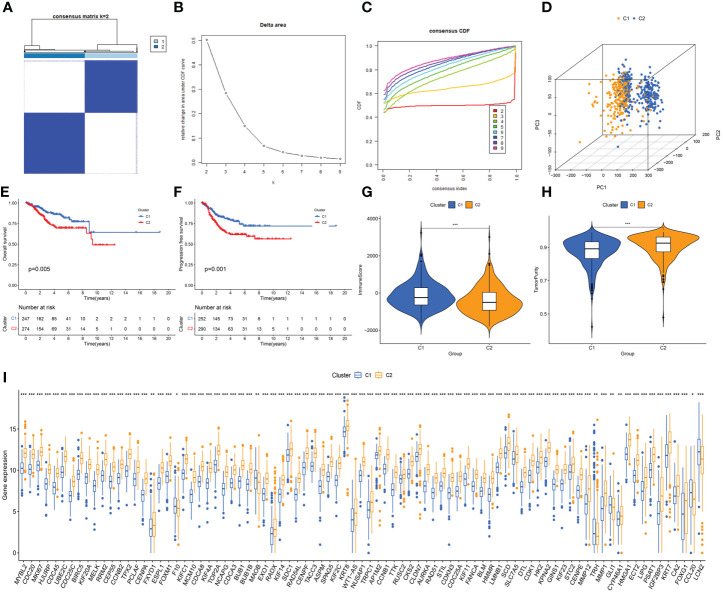
Unveiling ER stress-related subtypes through consensus clustering and subsequent analyses. **(A)** Consensus clustering matrix when k=2. **(B)** Relative alterations in CDF delta area curves. **(C)** Consensus CDF curves when k=2 to 9. **(D)** Three-dimensional Principal Component Analysis (3D PCA) delineating the segregation between Cluster C1 and Cluster C2. **(E, F)** The difference in overall survival and progress-free survival between the two clusters. **(G, H)** Correlations between the two clusters and immune scores, as well as tumor purity scores. **(I)** Histogram revealing the contrasting expression patterns of the 85 ERGs between the two clusters. *: p<0.05; **: p<0.01; ***: p<0.001.

Based on the expression of DEERGs, consensus clustering of 544 patients with UCEC in the TCGA cohort was performed. By gradually increasing the clustering variable (k) from 2 to 10, we observed the highest intragroup correlation at k=2, and relatively low intergroup correlations (**Figures 3A, B**). As k increased from 2 to 9, a significant change in the relative area under the cumulative distribution function (CDF) curve was observed (**Figure 3C**). This finding suggests that 544 patients with UCEC were effectively categorized into two clusters: C1 (n=252) and C2 (n=292). The PCA results revealed a pronounced separation of samples from these two clusters (**Figure 3D**), signifying substantial transcriptomic differences between them. The results of the survival analysis suggested that clusters C1 and C2 had significantly different prognostic outcomes, in which cluster C2 had a poorer prognosis and shorter OS and progression-free survival (**Figures 3E, F**).

Using the “estimate” algorithm, a tumor microenvironment (TME) score was obtained for each UCEC sample to understand the compositions of the TMEs in different clusters. In conclusion, the immunity score gradually decreased from C1 to C2 (**Figure 3G**), whereas tumor purity gradually increased (**Figure 3H**). Differential expression analysis revealed that 85 DEERGs were differentially expressed in different clusters, most of which were upregulated in cluster C2 (**Figure 3I**).

**Figure 4:**
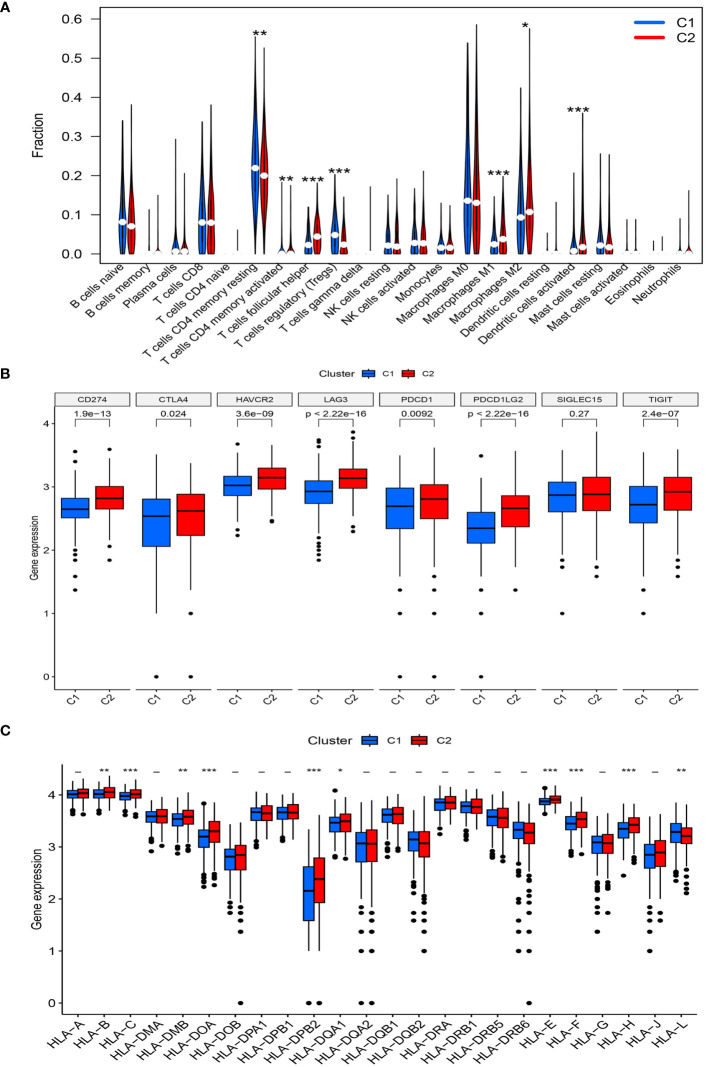
**(A)** The diagram of the difference in immune cell infiltration levels between the two clusters. **(B, C)** The expression level of immunosuppressive checkpoint genes and HLA-related genes, respectively (*, p < 0.05; **, p < 0.01; ***, p < 0.001).

Further analysis of immune cell infiltration in the two clusters using CIBERSORT revealed noteworthy increases in the infiltration levels of CD4 memory activated T cells, follicular helper T cells, M1 macrophages, M2 macrophages, and activated dendritic cells in cluster C2, compared with those in cluster C1. In contrast, the levels of CD4 memory resting T cells and regulatory T cells (Tregs) were decreased in cluster C2 (**Figure 4A**).

A high expression of most immune checkpoint genes (CD274, CTLA4, HAVCR2, TIGIT, LAG3, PDCD1, and PDCD1LG2) was observed in cluster C2, whereas SIGLEC15 expression was not significantly different between the two risk groups (**Figure 4B**). Meanwhile, the expression levels of HLA-B, HLA-C, HLA-DMB, HLA-DOA, HLA-B, HLA-DPB2, HLA-DQA1, HLA-E, HLA-F, and HLA-H significantly increased in cluster C2, whereas that of HLA-L decreased (**Figure 4C**).

### 3.3 Identification of hub DEERGs

**Figure 5:**
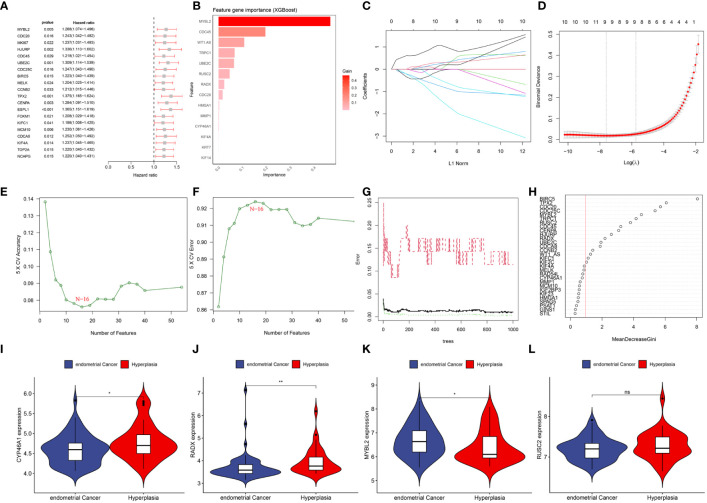
**(A)** Top 20 genes associated with overall survival via univariate COX analysis. **(B)** Screening diagnostic biomarkers based on the XGBoost algorithm(n=14). **(C, D)** The variables selection in the LASSO model(n=9). **(E, F)** Optimal biomarkers screening by SVF-RFE algorithm(n=16). **(G, H)** Significant feature selected via the random forest algorithm(n=21). **(I–L)** The expression validation between control and UCEC based on GSE106191. **(I)** CYP46A1; **(J)** RADX; **(K)** MYBL2; **(L)** RUSC2. ns: p>0.05; *: p<0.05; **: p<0.01.

By combining the UCEC expression profiling data and clinical information, a group of 521 cancer patients was obtained from the TCGA-UCEC cohort of 579 patients with a survival time of >30 days. Using univariate Cox regression analysis, 54 DEERGs that were significantly associated with prognosis were identified in the TCGA-UCEC cohort (**Figure 5A**). Additionally, using machine learning algorithms, the key genes from the 54 DEERGs were identified. The XGBoost algorithm further identified 14 hub genes (**Figure 5B**). In the LASSO regression analysis, an optimal lambda value of 0.0005 was determined after ten cross-validations, and nine key genes were identified (**Figures 5C, D**). During the SVM-RFE, the classifier error was minimized when the number of features was 16. These 16 genes were identified as hub genes (**Figures 5E, F**). The random forest algorithm identified 21 DEERGs with importance scores of >1.0 as hub features (**Figures 5G, H**). Overall, CYP46A1, RADX, MYBL2, and RUSC2 overlapped among the four machine learning algorithms and were recognized as hub DEERGs. Details of the results from these machine learning algorithms are provided in **Supplementary Table 2**. In the external validation set GSE106191, the expression levels of CYP46A1 and RADX were downregulated, while that of MYBL2 was upregulated. No significant difference in the expression level of RUSC2 was identified (**Figures 5I–L**).

### 3.4 Establishment and verification of the risk score model

**Figure 6:**
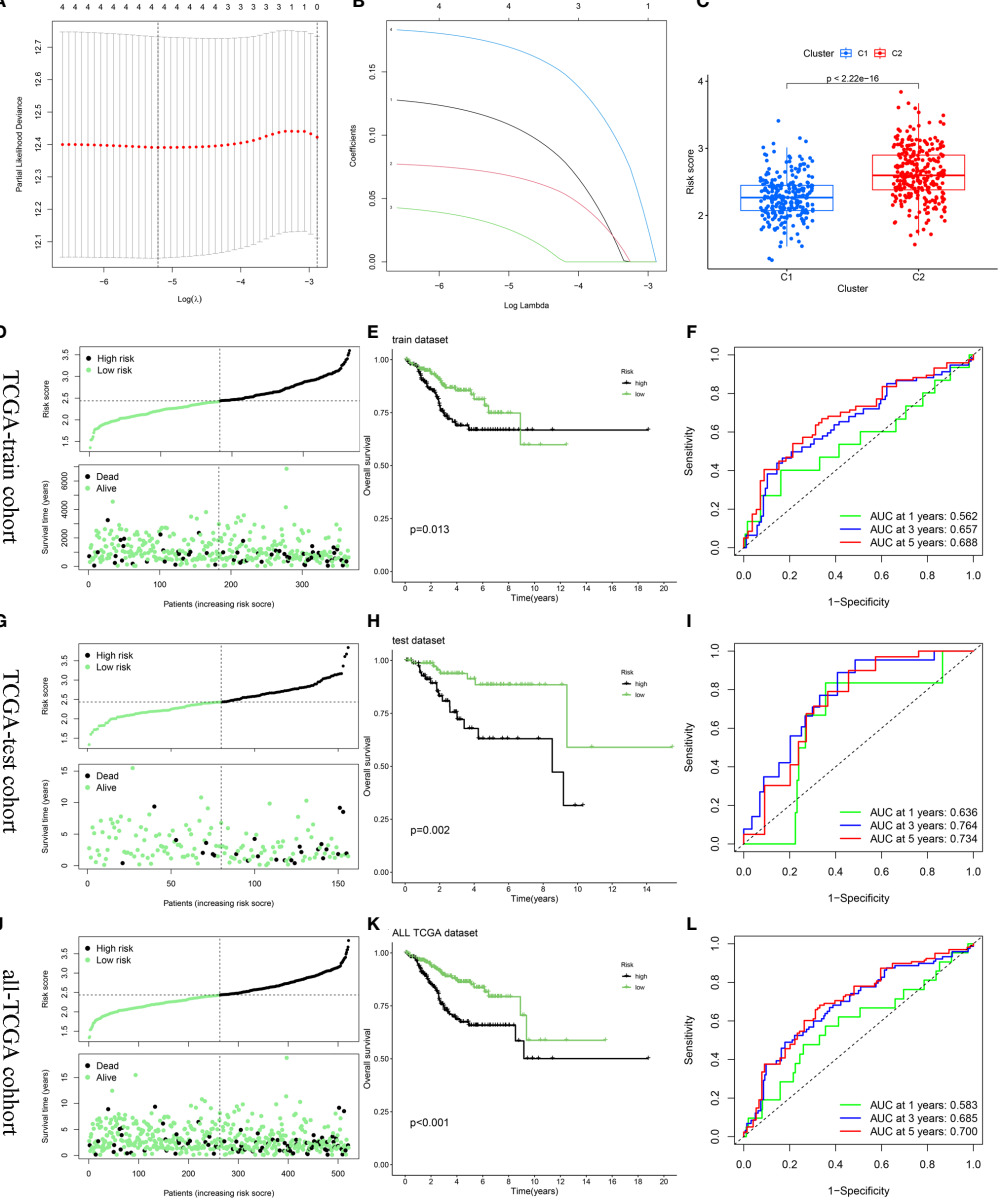
Construction and validation of the risk score model. **(A, B)** Constructed a prognostic model in the TCGA-train cohort through LASSO COX regression analysis. **(C)** The difference in risk scores between the two ERGs subtypes. **(D, G, J)** Risk scores distribution and survival status of each patient in the TCGA-train cohort, TCGA-train cohort, and all-TCGA cohort, respectively. **(E, H, K)** Kaplan–Meier curves for the OS of the two subtypes in the TCGA-train cohort, TCGA-train cohort, and all-TCGA cohort, respectively. **(F, I, L)** ROC curves illustrated the predictive efficacy of the risk score for 1-, 3-, and 5-year survival in the TCGA-train cohort, TCGA-train cohort, and all-TCGA cohort, respectively.

After random partitioning, TCGA-train and TCGA-test cohorts comprised 355 and 156 patients, respectively. A prognostic model was constructed for the TCGA-train cohort based on the four hub DEERGs (**Figures 6A, B**). The risk score was calculated as follows: riskscore = (0.113 × MYBL2 expression) + (0.070 × RADX expression) + (0.030 × RUSC2 expression) + (0.173 × CYP46A1 expression). Patients in the TCGA-train, TCGA-test, and all-TCGA cohorts were stratified into high- and low-risk groups according to the median risk score. Risk scores in different clusters constructed based on 94 DEERGs were analyzed, suggesting that cluster C2 exhibited a significantly higher risk score than cluster C1 (**Figure 6C**).

The relationship between risk score and survival status was also explored. Scatter plots showed that patient mortality increased with increasing risk score (**Figure 6D**). Kaplan-Meier analyses were used to determine the prognostic value of the risk model. Overall, high-risk scores were associated with unfavorable OS in the TCGA-train cohort (**Figure 6E**). To further underscore the prognostic accuracy of the model, a time-dependent ROC analysis was conducted. The area under the ROC curve (AUC) values for the TCGA-train cohorts at 1, 3, and 5 years were 0.562, 0.657, and 0.688, respectively (**Figure 6F**).

To validate the accuracy and stability of the model, the same analysis was conducted for both the TCGA-test and all-TCGA cohorts. The results indicated a worse prognosis in the high-risk group than that in the low-risk group (**Figures 6G, H, J, K**). In the TCGA-test cohort, the AUC values at 1, 3, and 5 years were 0.636, 0.764, and 0.734, respectively (**Figure 6I**). In the all-TCGA cohort, the corresponding AUC values were 0.583, 0.685, and 0.700 at 1, 3, and 5 years, respectively (**Figure 6L**).

### 3.5 Construction and validation of a predictive nomogram

**Figure 7:**
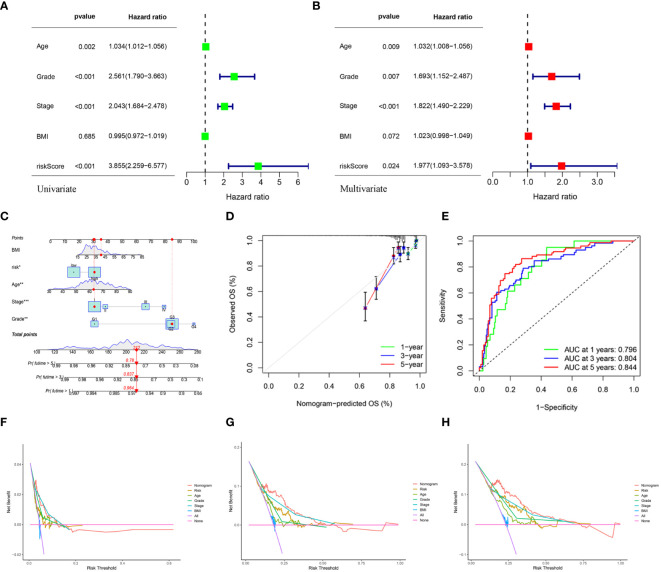
**(A, B)** Univariate and multivariate analyses showed the prognostic value of the clinical features and risk scores. **(C)** The construction of the nomogram. **(D)** Calibration curves to evaluate the performance of the nomogram for 1-, 3-, and 5-year OS, respectively. **(E)** The AUC of the nomograms compared for 1-, 3-, and 5-year OS, respectively. **(F–H)** The DCA curves of the nomogram for 1-, 3-, and 5-year OS in TCGA-UCEC, respectively.

Univariate and multivariate Cox regression analyses indicated that age, grade, tumor stage, and risk score were independent risk factors of UCEC. An elevated risk score was independently associated with poor OS, suggesting that it was an independent prognostic factor in patients with UCEC (**Figures 7A, B**). The clinical features and risk scores were integrated to construct a nomogram (**Figure 7C**) for individualized prediction of the 1-, 3-, and 5-year survival probabilities of patients with UCEC. The calibration plot demonstrated high consistency between the observed and predicted values (**Figure 7D**). Analysis of the calibration curves provided a more comprehensive understanding of the model’s performance and ensured its validity in clinical practice. Based on the nomogram, the AUC values of the 1-, 3-, and 5-year ROC curves for OS prediction were 0.796, 0.804, and 0.844, respectively (**Figure 7E**). The DCA results indicated that the nomogram offered optimal clinical net benefit for 3- and 5-year OS, although it did not provide the optimum clinical net benefit for 1-year OS (**Figures 7F–H**). These findings suggest that a nomogram based on risk score can serve as an effective prognostic prediction tool in clinical practice.

### 3.6 Verification of the expression of hub DEERGs

**Figure 8:**
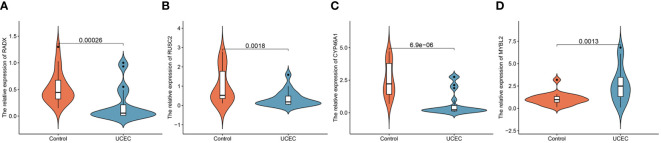
The mRNA expression validation between control and UCEC tissues by RT-qPCR. **(A)** RADX; **(B)** RUSC2; **(C)** CYP46A1; **(D)** MYBL2.

**Figure 9:**
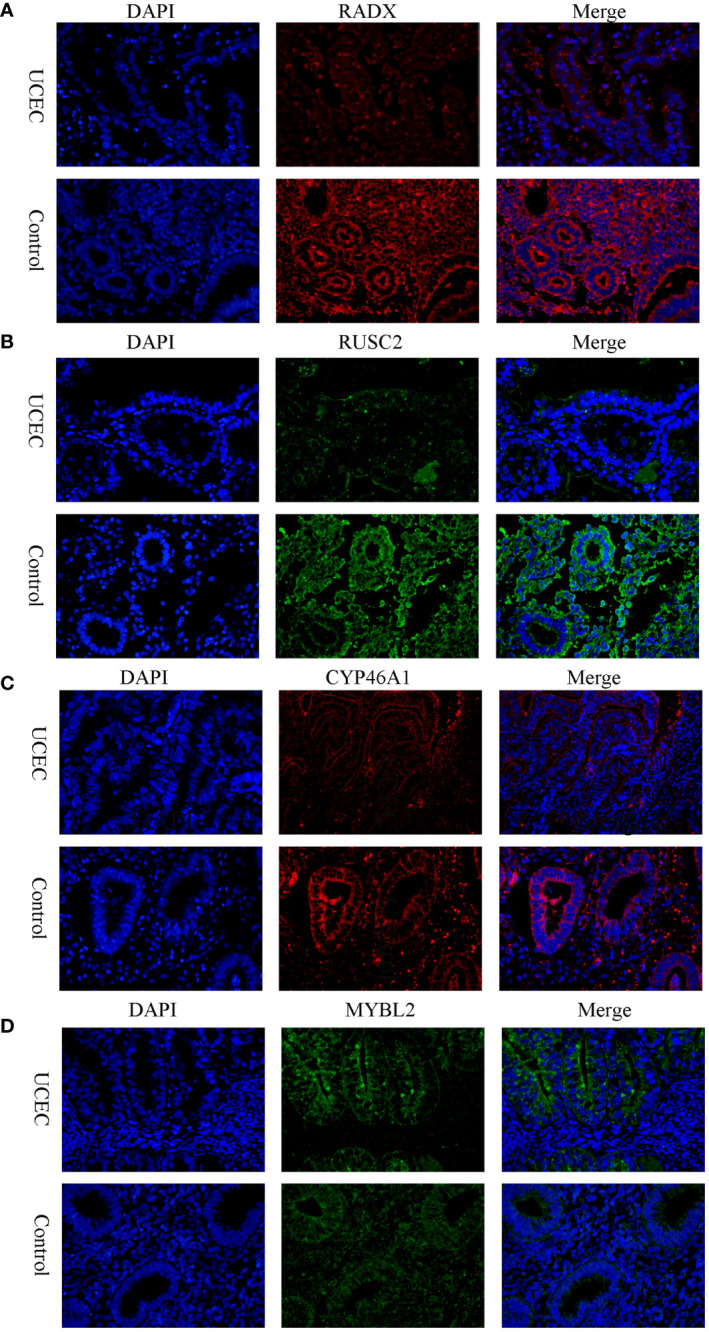
The protein expression level of RADX, RUSC2, CYP46A1, and MYBL2 between UCEC and control tissues by immunofluorescence. **(A)** RADX; **(B)** RUSC2; **(C)** CYP46A1; **(D)** MYBL2.

Clinical tissues were collected from 20 control patients and 20 patients with UCEC to verify the mRNA and protein expression of the hub genes. The RT-qPCR results showed that the relative expressions of RADX, RUSC2, and CYP46A1 in the UCEC group were lower than those in the control group, whereas the relative expression of MYBL2 mRNA was upregulated (**Figures 8A–D**). Immunofluorescence analysis revealed that the protein expression levels of the four hub genes were consistent with the RT-qPCR results (**Figures 9A–D**).

### 3.7 Potential drugs related to UCEC

**Figure 10:**
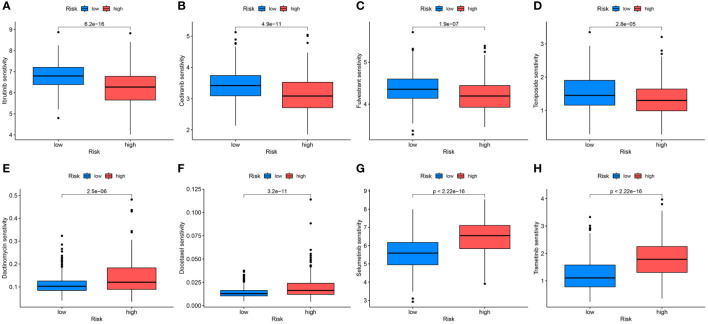
Differential analyses of IC50 values of common drugs between the high- and low-risk groups. **(A)** ibrutinib; **(B)** cediranib; **(C)** fulvestrant; **(D)** teniposide; **(E)** dactinomycin; **(F)** docetaxel; **(G)** selumetinib; **(H)** trametinib.

Differences in the IC50 levels of chemotherapeutic agents between the high- and low-risk groups were further investigated. The results indicated significantly lower IC50 values for ibrutinib, cediranib, fulvestrant, and teniposide in the high-risk group than those in the low-risk group. This suggests that patients with high-risk scores could potentially benefit more from the use of these drugs (**Figures 10A–D**). Conversely, in the low-risk group, dactinomycin, docetaxel, selumetinib, and trametinib may offer greater clinical benefits (**Figures 10E–H**).

## 4 Discussion

UCEC is the most prevalent gynecological disease in developed countries. Disease-free survival (DFS) in patients with UCEC is influenced by factors such as age, menopausal status, tumor stage, grade, and lymphovascular gap invasion. Despite advancements in our understanding and management of endometrial cancer, variability in patient outcomes, especially among patients with advanced-stage disease or recurrence, remains a significant challenge. This variability stems in part from the limitations of traditional prognostic markers and the one-size-fits-all approach to treatment, which fail to account for the molecular complexity and diversity of endometrial cancer. Standard treatment modalities, while effective for a subset of patients, fall short of providing targeted therapeutic options for patients with high-risk disease features or those who develop resistance to conventional treatments. Therefore, refined prognostic tools and tailored therapeutic strategies are urgently required. Recent studies based on public databases, such as TCGA and GEO, have identified specific genes associated with UCEC tumor prognosis and treatment response. For instance, polymerase-epsilon (POLE)-mutant tumors demonstrate significantly improved progression-free survival, whereas tumors with high copy numbers exhibit worse prognoses (26). The emergence of molecular subtyping-based immunotherapy has presented a promising therapeutic avenue for advanced and recurrent UCEC and has demonstrated notable efficacy (27). The management of metastatic UCEC poses a significant challenge, particularly following failure of first-line chemotherapy. Therefore, exploration of new prognostic biomarkers and development of more precise prognostic models to guide treatment strategies for UCEC are crucial.

The role of ER stress in cancer has garnered substantial interest because of its pivotal role in promoting tumor growth, proliferation, metastasis, invasion, angiogenesis, and chemotherapy resistance (28). Uncontrolled tumor cell growth, hypoxia, nutrient deprivation, oxidative stress, and DNA damage in tumors can result in ER stress. ER stress induces an unfolded protein response, enabling tumor cell adaptation for survival. It also leads to the release of proinflammatory factors and the reprogramming of immune cell function, thereby promoting tumor progression and immune escape (29, 30). Numerous studies have identified ERGs as valuable predictors of cancer prognosis. For instance, Zhang Q identified 16 ERGs that offer insights into the immune profile of gliomas and provide a basis for prognostic assessments (31).

In hepatocellular carcinoma, Song D verified the differential expression of 6 ERGs through *in vitro* experiments, shedding light on their potential roles in tumor dynamics (32). Furthermore, ERGs have been reported in clear cell renal cell carcinoma and pancreatic ductal adenocarcinoma, demonstrating their broad relevance to different cancer types (33, 34). Given these insights, targeting ER stress and its signaling pathways is a promising strategy for cancer therapy. Inhibitors of unfolded protein response components, such as PERK and IRE1α, are being explored for their potential to sensitize cancer cells to chemotherapy and radiotherapy by exacerbating ER stress, leading to cell death (35, 36). Additionally, the modulation of ER stress responses to enhance antitumor immunity is an emerging area of research that offers new avenues for combination therapies to improve clinical outcomes (37).

Several studies have investigated the association between ER stress and UCEC. The activation of the unfolded protein response and increased expression of GRP78 following ER stress may promote UCEC growth and invasion (11). Furthermore, GRP78 knockdown significantly decreases Ishikawa cell growth, suggesting a direct association between ER stress and UCEC progression (38).

Zhou and Zhang (39, 40) also analyzed TCGA-UCEC data and created a prognostic model. While both our study and theirs rely on bioinformatic data analysis, our research appears to be more comprehensive and in-depth. First, we analyzed multiple UCEC datasets from different databases to enhance the generalizability of our results. Furthermore, the adoption of advanced algorithms, including WGCNA and machine learning techniques, facilitated the targeted identification of crucial hub genes. The incorporation of *in vitro* validation techniques such as RT-PCR and immunofluorescence in clinical UCEC specimens reinforced the results of our study.

In the present study, a prognostic model based on the investigation of ER stress-related genes was constructed using sophisticated bioinformatics analyses. This approach represents a significant step forward in addressing the challenges posed by endometrial cancer. By investigating the molecular intricacies of endometrial cancer, particularly the role of ER stress in tumorigenesis and disease progression, our model offers a more nuanced understanding of the biological underpinnings of endometrial cancer.

Building on our initial findings, this comprehensive analysis of multiple endometrial cancer datasets from TCGA and GEO databases identified 94 significant DEERGs. By leveraging these genes, we stratified the TCGA-UCEC dataset into two distinct clusters, revealing that patients in cluster C2 exhibited significantly worse OS and progression-free survival than those in cluster C1. This discovery not only demonstrates the feasibility of stratifying patients with UCEC based on DEERGs but also highlights the increased risk associated with cluster C2.

Further in-depth analysis suggested that the observed survival disparity between clusters may be linked to the unique immunoenvironmental characteristics of cluster C2. Specifically, increased immune cell infiltration, activation of immunosuppressive signaling pathways, and aberrant HLA gene expression in cluster C2 collectively contributed to the observed adverse prognosis. The simultaneous overactivation and immunosuppression within cluster C2 suggests a complex mechanism that allows cancer cells to evade immune surveillance. These insights emphasize the importance of considering the unique immunogenetic landscape of patients with endometrial cancer in the development of personalized treatment strategies. However, the findings presented herein require further validation through laboratory studies and clinical trials. Future research should focus on elucidating the specific roles of these DEERGs in the development of endometrial cancer and how they modulate immune responses and promote immune escape.

Using Cox regression analysis, we successfully identified 54 DEERGs. To identify critical genes within this extensive list, we utilized a sophisticated array of machine learning algorithms: LASSO, XGBoost, SVM-RFE, and Random Forest. LASSO introduced an L1 regularization term that facilitated automatic feature selection by narrowing certain model coefficients to zero. XGBoost, a gradient-boosting framework, synergized the strengths of decision trees and gradient boosting, iteratively enhancing model performance. SVM-RFE progressively trimmed the feature set by iteratively training the support vector machine, eliminating features with minimal contributions to classification to reveal key features. Random Forest, which utilizes multiple decision trees, performed classification or regression and enhanced model stability and accuracy through voting or averaging. Employing these algorithms allowed us to distill the 54 DEERGs into four key hub genes: MYBL2, RADX, RUSC2, and CYP46A1.

MYBL2 belongs to the MYB family of transcription factors, which perform crucial physiological regulatory functions in cell cycle progression, survival, and differentiation. Research has indicated that elevated MYBL2 expression in various tumors is linked to unfavorable prognoses, as observed in bladder cancer and hepatocellular carcinoma. Specifically, MYBL2 contributes to the proliferation and metastasis of bladder cancer by upregulating the expression of cell division cycle-associated protein 3 (CDCA3) (41). Additionally, a study by Frau showed that increased expression of MYBL2 in hepatocellular carcinoma actively contributed to the biological progression of tumors by influencing the cell cycle (42).

RADX is an X-linked single-stranded DNA-binding protein related to RPA1 and is essential for maintaining genome stability. Although little research has been conducted on RADX in cancer, evidence suggests that inactivation of RADX may confer resistance to chemotherapy and PARP inhibitors in cancer cells (43).

CYP46A1 (cytochrome P450 family 46 subfamily A member 1) plays a crucial role in drug metabolism and lipid synthesis. In glioblastoma, the expression of CYP46A1 is significantly reduced and correlates with tumor grade and prognosis. Its ectopic expression reduces the proliferation and invasive capacity of glioblastoma multiforme cells (44). Moreover, CYP46A1 transcripts are overexpressed in certain human pancreatic neuroendocrine tumor samples, correlating with tumor diameter (45). CYP46A1 has been identified as a promoter of colorectal cancer progression by inducing tumor cell proliferation and angiogenesis (46).

RUSC2 is a less investigated multi-structural domain protein that is functionally associated with the Rap and Rab GTPase families. Mutations in RUSC2 in neuroblastoma have been identified as potential drivers (47). Duan has demonstrated that RUSC2 is commonly expressed in diverse lung cancer cells, knockdown of RUSC2 effectively inhibits the migration of lung cancer cells, and RUSC2 regulates the progression of lung cancer through epidermal growth factor receptor (EGFR) signaling (48).

By constructing a prognostic model based on the four hub DEERGs and categorizing patients into distinct risk groups within the TCGA-train cohort, we unveiled a stark contrast in mortality rates, with the high-risk group exhibiting significantly elevated mortality. The prognostic model’s validity, confirmed across the UCEC-test and all-TCGA cohorts, underscores the robustness and universal applicability of our findings. The stratification of patients based on risk scores, particularly the pronounced disparity between clusters C1 and C2, emphasizes the critical role of MYBL2, RADX, RUSC2, and CYP46A1 in molecular stratification and prognostic forecasting in UCEC. This stratification not only provides a deeper understanding of UCEC molecular diversity but also has substantial implications for personalized treatment approaches.

The development of a robust prognostic model rooted in ER stress-related genes through exhaustive bioinformatic analysis is a promising advancement. However, the retrospective nature of our study, which relied heavily on existing TCGA and GEO database information, introduces inherent limitations to our conclusions. Although our initial experimental validations confirmed the differential expression of the key hub genes, future research must expand into more comprehensive cellular and animal studies. Such endeavors are vital to elucidate the precise biological functions of these ERGs and to decode the complex mechanisms by which they influence the pathogenesis and progression of UCEC.

In this study, WGCNA was used to screen 94 DEERGs, and patients with UCEC were categorized into two subtypes based on these genes, with cluster C2 patients having a poorer prognosis. Using Cox regression modeling and machine learning algorithms, RADX, RUSC2, CYP46A1, and MYBL2 were identified as diagnostic and prognostic markers for UCEC. A prognostic model established using these four genes could effectively evaluate patient prognosis and guide clinical decisions. In summary, this study contributes significantly to the field by enhancing the molecular understanding of endometrial cancer, introducing methodological innovations, advancing personalized medicine through prognostic modeling, and highlighting new therapeutic targets.

## Data availability statement

Publicly available datasets were analyzed in this study. This data can be found here: https://portal.gdc.cancer.gov/projects/TCGA-UCEC. https://www.ncbi.nlm.nih.gov/geo/query/acc.cgi(GSE17025, GSE63678, GSE115810, and GSE106191).

## Ethics statement

The studies involving humans were approved by the Research Ethics Committee of the First Affiliated Hospital of Guangxi Medical University. The studies were conducted in accordance with the local legislation and institutional requirements. The participants provided their written informed consent to participate in this study.

## Author contributions

SL: Conceptualization, Data curation, Software, Validation, Writing – original draft, Writing – review & editing. CW: Data curation, Software, Visualization, Writing – original draft. YW: Software, Validation, Visualization, Writing – original draft. JF: Conceptualization, Funding acquisition, Supervision, Writing – review & editing.
